# An Evaluation of the Interaction of Brefeldin A with Mitogen-Activated Protein Kinase 1 (MAPK1) and Protein Kinase C Alpha (PrKCα): Insights from Molecular Modelling Studies

**DOI:** 10.3390/ijms27073240

**Published:** 2026-04-02

**Authors:** Vivash Naidoo, Ikechukwu Achilonu, Marushka Soobben, Emmanuel Iwuchukwu, Nikita Singh, Jeyalakshmi Kandhavelu, Rodney Hull, Sheefa Mirza, Clement Penny

**Affiliations:** 1 Department of Internal Medicine, Faculty of Health Sciences, University of the Witwatersrand, Johannesburg 2193, South Africa; vivash.naidoo@wits.ac.za; 2 Department of Family Medicine and Primary Health Care, University of the Witwatersrand, Johannesburg 2193, South Africa; 3 Protein Structure-Function Research Unit, School of Molecular and Cell Biology, Faculty of Science, University of the Witwatersrand, Johannesburg 2050, South Africa; ikechukwu.achilonu@wits.ac.za (I.A.); marushka.soobben@wits.ac.za (M.S.); emmanuel.iwuchukwu@wits.ac.za (E.I.); 4 Department of Microbiology, School of Molecular and Cell Biology, Faculty of Science, University of the Witwatersrand, Johannesburg 2050, South Africa; nikita.nankoo@wits.ac.za; 5 Department of Oncology, Lombardi Comprehensive Cancer Centre, Georgetown University Medical Centre, Washington, DC 20007, USA; biojeyaa@gmail.com; 6 Pan African Research Institute, University of Pretoria, Pretoria 0002, South Africa; rodney.hull@up.ac.za

**Keywords:** colorectal cancer, glycosylation, Brefeldin A, MAPK1, PrKCα, molecular docking, protein inhibition

## Abstract

Aberrant protein glycosylation is a key driver of colorectal cancer (CRC) progression, contributing to tumour growth, metastasis, and immune evasion. In this study, computational approaches were employed to explore the potential of Brefeldin A as an inhibitor of two glycosylation-associated regulatory proteins: Protein Kinase C alpha (PrKCα) and Mitogen-Activated Protein Kinase 1 (MAPK1). Using computational docking and structural analyses, Brefeldin A was predicted to bind effectively to both targets, thereby inhibiting their enzymatic activities. Detailed investigations revealed that Brefeldin A interacts favourably within the active sites of MAPK1 and PrKCα, forming stable complexes by optimal binding interactions. Key residues contributing to binding stabilisation were identified in both MAPK1 and PrKCα. For MAPK1, residues such as Lys114 and Ser153 played a significant role in hydrogen bonding interactions, while for PrKCα, Gln105, Asn154, and Asp167 were notably involved. These interactions included both hydrogen bonds and hydrophobic contacts, which collectively contributed to the strength and specificity of ligand binding. The identification of these residues provides insight into the molecular mechanisms underlying the stabilisation of the Brefeldin A-kinase complexes. Binding affinity estimations showed that Brefeldin A bound to MAPK1 exhibited a binding energy of −22.18 ± 4.50 kcal/mol. In contrast, the Brefeldin A bound to PrKCα demonstrated a slightly stronger binding energy of −23.90 ± 5.36 kcal/mol. Collectively, these findings underscore Brefeldin A’s potential as a novel inhibitor targeting glycosylation-related proteins in CRC, offering a promising therapeutic strategy to impede CRC progression. This work not only proposes Brefeldin A as a promising therapeutic lead but also supports glycosylation inhibition as a valuable approach for CRC control, with broader implications for drug discovery in glycan-related oncogenic pathways.

## 1. Introduction

Protein glycosylation is an intricate and significant post-translational modification (PTM) that controls the biological activities and functions of glycoproteins [1]. This primary PTM occurs within the endoplasmic reticulum/Golgi compartment of all cells [2,3]. This process is coordinated by a series of glycosyltransferase and glycosidase enzymes that eventually produce different carbohydrate structures (glycans) [2,3,4]. In the investigation of cancer progression, pathological changes during glycosylation are considered a hallmark of cancer [2,4]. Glycosylation is essential for recognition, signalling, and interaction events that occur in both inter- and intracellular proteins. These events have crucial structural roles in the folding, conformation, trafficking, and degradation of these glycoproteins [3,5]. Additional roles played by glycosylation include, but are not limited to, adhesion, cell–matrix interactions, protection against proteases and immune recognition, and membrane organisation [2]. Glycans mediate fundamental molecular, cellular, tissue, organ, and systemic biological processes responsible for normal physiological functions, implicating them in numerous human diseases, including cancer [6]. Hence, slight changes in carbohydrate structure can profoundly affect biological cell processes, resulting in oncogenic transformation [2]. Changes in the glycosylation profiles of membrane proteins are characteristic of many cancer cell types, serving not only as potential biomarkers for cancer, but also as factors that influence tumorigenesis and metastasis [7]. Considering the general roles of glycosylation, it can be inferred that novel targets in the field of glycobiology, such as glycoforms and glycosyltransferases involved in glycans synthesis, could have the potential for drug discovery.

While aberrant protein glycosylation is a common feature of many cancers, the gut has a particularly rich glycoprotein landscape. This is due to the walls of the gut being heavily coated in glycoproteins such as mucins. This means that the function of the epithelial barrier and its role in microbiota interactions and gut inflammation relies on protein glycosylation. Additionally, many biomarkers used in clinical diagnosis and monitoring in CRC are glycoproteins [8]. *N*-glycosylation patterns can distinguish CRC from normal tissue and carry prognostic information [9]. Marked shifts in *N*-glycan composition have been identified in CRC tissue compared to adjacent normal tissue. These shifts are linked to increased high-mannose *N*-glycans and decreased complex, sialylated, and fucosylated *N*-glycans [10]. These changes are also associated with a dysregulated expression of multiple glycosyltransferases [8].

Previous studies have suggested that Protein Kinase C alpha (PrKCα) and mitogen-activated protein kinase 1 (MAPK1) are involved in glycosylation [11,12,13,14]. PrKCα and MAPK1 have been reported extensively to regulate glycosylation and are viable therapeutic targets. PrKCα is a serine/threonine kinase family member essential in signal transduction [15], and plays a role in cell proliferation, differentiation, survival, invasion, migration, apoptosis, angiogenesis, and drug resistance [16]. Their significant contribution to cell signalling makes them promising therapeutic targets for several diseases, including various cancers [16]. Studies have revealed that PrKCα is essential for neoplastic transformation, carcinogenesis and tumour cell invasion; therefore, these findings have presented PrKCα as a potential target for anticancer therapy [15]. Different cellular responses are produced by PrKCα activation, which is responsible for a diverse set of responses, including the expression of growth factors, activation of signalling pathways and response to oxidative stress [17]. Several obtainable catalytic and regulatory mechanisms have suggested that PrKCα has significant potential for targeted drug design [18,19,20].

MAPK1 cascades play a crucial role in environmental signal transduction and stress-induced defence responses; these signals are adversely regulated by specific Ser/Thr protein phosphatases of the type 2C (PP2C) and dual-specificity phosphatase (DSP) families, which are responsible for inactivating stress-induced MAPKs [21]. The MAPK1 signalling pathway cascade comprises three kinases: extracellular signal-regulated kinase (ERK), c-Jun N-terminal kinase (JNK), and p38. Here, the upstream kinase (MAPKKK) responds to diverse extra- and intracellular signals and further activates the middle kinase (MAPKK) via a direct phosphorylation reaction [22]. The entire process results in MAPK phosphorylation by MAPKKs, leading to physiological conditions such as cell proliferation, differentiation, survival, and death [23]. Counteractions and reactions of protein kinases and protein phosphatases, as applied in reversible protein phosphorylation, are among the mechanisms exploited by MAPK1 to transmit stress and developmental signals at the molecular level [24,25]. Findings have suggested a dual role for MAPK1 in cancer growth prevailing in specific cases and circumstances [26]. An increase in MAPK1 phosphorylation has been reported to be highly associated with malignancy in particular types of cancers [27,28,29]. MAPKs can be dephosphorylated and thus inactivated by specific protein phosphatases; thus, attenuation of MAPKs can serve as an excellent therapeutic approach and can be exploited for drug discovery. MAPK1 is not unique to CRC, but the MAPK/ERK pathway is one of the core oncogenic signalling axes in colorectal tumourigenesis, activated downstream of EGFR, RAS, and RAF. EGFR–RAS–RAF–MEK–MAPK1/3 is a core proliferative pathway in CRC progression, activated by growth factors and contributing to cell proliferation, survival, angiogenesis, and invasion [30]. While not being uniquely overexpressed in CRC, its pathway activation is a hallmark of CRC biology, a pathway which converges on MAPK1/ERK2 activation. Therefore, it is a valid therapeutic target. [31]. PRKCA is not CRC-specific, but it plays documented roles in colon epithelial transformation, including epithelial barrier integrity, inflammation-associated carcinogenesis, tumour progression, and signalling crosstalk. It fulfils this last function by modulating EGFR signalling and MAPK pathway activation [31].

Small molecules that can inhibit glycosylation enzymes or the cellular glycosylation process have been identified as potential drug candidates in the therapeutic advancement toward treating cancer [2,32]. Brefeldin A, a fungal metabolite derived from Penicillium Brefeldianum, is an inhibitor that swiftly blocks protein transport from the endoplasmic reticulum (ER) to the Golgi network. It carries out this process by affecting protein glycosylation by preventing COP-I assembly and access to emerging ER glycoproteins to the Golgi-located glycosylation process [2,33,34,35]. While Brefeldin A’s effects on intracellular trafficking are well-established, its molecular interaction with glycosylation-associated kinases remains uncharacterised.

Therefore, this study aims to employ computational techniques to elucidate the molecular mechanisms underlying the potential inhibitory interactions between Brefeldin A and the key kinases MAPK1 and PrKCα. By exploring these interactions, the study seeks to identify novel insights into kinase-mediated glycosylation regulation and to evaluate Brefeldin A’s potential as a dual inhibitor and repurposed therapeutic candidate not only for CRC but also for various other cancers.

## 2. Results

### 2.1. Screening Glycosylation Targets

The SMILES (Simplified Molecular-Input Line-Entry System) chemical structure of Brefeldin A was used in Swiss Target Protein Prediction to identify potential biological targets. The canonical SMILES structure of Brefeldin A was obtained from the PubChem database, and this standardised format served as input for various computational tools for target prediction. This process allowed systematic evaluation of the binding affinity of Brefeldin A to various proteins. Table 1 lists proteins that we identified as being potential targets for further study and drug development using SwissTargetPredict (2019 version).

Swiss Protein Predict (2019 version) is an in silico protein target prediction software that helps identify potential protein targets for a given compound by analysing the compound’s structure and evaluating its interactions with proteins. Table 1 illustrates the protein targets for Brefeldin A and their respective known actives in 3D and 2D, as predicted by the Swiss Protein Predict tool. Known actives in 3D and 2D provide insight into the potential interactions and binding affinities between Brefeldin A and the protein targets. A higher number of known actives suggests that the compound may have a stronger affinity for the specific protein target and, thus, a higher likelihood of modulating its function [36].

Two proteins of interest were identified as potential targets by manual curation, and PrKCα and MAPK1 were selected, with KEGG pathway analysis showing their involvement in various cellular signalling pathways that regulate essential processes, such as pathways in cancer, proteoglycans in cancer, MAPK1 signalling, and AGE-RAGE signalling pathways, to name a few. Some of the critical pathways to note are shown in Table 2. The genes highlighted in yellow are the specific genes of interest which were further investigated.

### 2.2. Brefeldin Docking and MM/GBSA-Based HTVS

A total of 43 brefeldin analogues were initially subjected to the high-throughput virtual screening (HTVS) workflow described in the methods. The full ligand set was first evaluated using Glide HTVS and SP docking, after which compounds were filtered using QikProp-predicted physicochemical and ADME properties to remove analogues with poor drug-likeness, violations of Lipinski/Veber criteria, or predicted liabilities. The remaining ligands were then ranked according to their docking scores, key residue interactions, and pose quality. From this ranked list, the top-scoring subset was further refined using Prime MM/GBSA binding free-energy calculations. Table 3, therefore, represents the final set of ligands that passed all filtering steps, docking score thresholds, QikProp property filters, and MM/GBSA rescoring, and not the entire 43-compound library. The analogue discussed in detail below corresponds to the highest-ranked hit emerging from this multistep selection pipeline.

**Figure 1:**
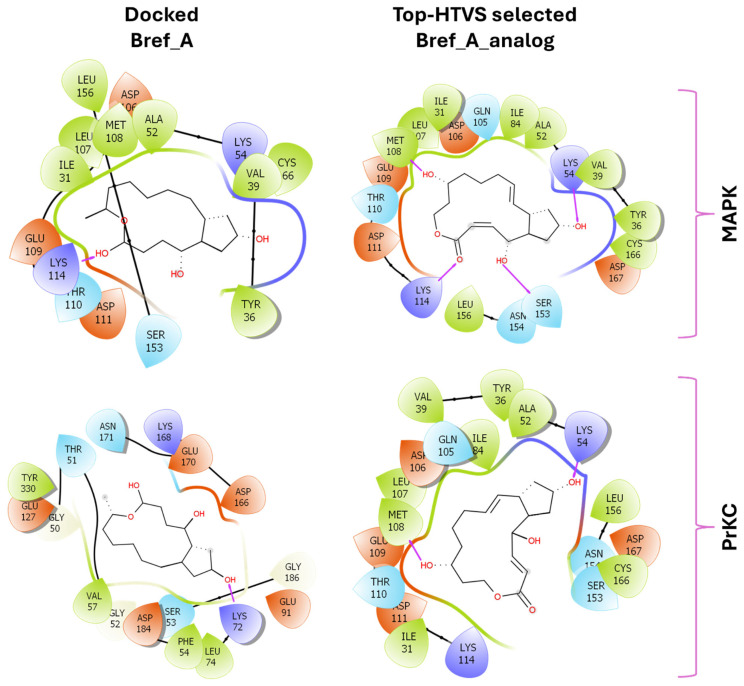
Two-dimensional docking interaction diagrams of Brefeldin A and its analogue in MAPK1 and PrKCα: The panels illustrate the binding interactions of Brefeldin A (**left**) and its structural analogue (**right**) within the active sites of MAPK1 (**top**) and PrKCα (**bottom**). Each ligand is shown in stick representation at the centre of the binding pocket, surrounded by amino acid residues. Hydrogen bonds are indicated by magenta arrows, while hydrophobic and van der Waals contacts are represented by coloured residue shells. Negatively charged residues (Asp, Glu) are coloured red; positively charged residues (Lys, Arg) are blue; polar residues are light purple; and hydrophobic residues (Leu, Ile, Val, Met, Ala, etc.) are green.

As shown in Figure 1, Brefeldin A and its top-ranked analogue exhibit distinct binding profiles within the active sites of MAPK1 and PrKCα. In MAPK1, Brefeldin A forms limited hydrogen-bond interactions, primarily with LYS114 and SER153. In contrast, the analogue engages a broader set of residues, including GLN105, ASN154, and ASP167, resulting in an expanded network of hydrogen-bonding and hydrophobic contacts. These residues in MAPK1 are located within the ATP-binding cleft and adjacent regulatory regions of the kinase domain and thus contribute directly to ligand stabilisation within the active site. These residues are not associated with glycosylation-related kinase activity; rather, they participate in canonical MAPK1 catalytic-site interactions that govern ATP positioning, substrate recognition, and small-molecule binding. A similar trend is observed in PrKCα: Brefeldin A establishes only a few stabilising interactions, whereas the analogue interacts with key catalytic-site residues such as ASP166, GLU170, and LYS168. This more extensive interaction network suggests improved stabilisation of the analogue within both kinase binding pockets, consistent with the study’s aim of identifying structural modifications that enhance affinity and selectivity.

The Prime MM/GBSA binding free-energy decomposition further supports the docking-based observations (Table 3). For MAPK1, the analogue displays favourable binding free energies (Δ*G*_bind_ = −42.33 to −33.70 kcal/mol), driven largely by strong van der Waals (Δ*G*_vdW_ = −29.57 to −37.57 kcal/mol) and Coulombic contributions (Δ*G*_coulomb_ = −11.27 to −24.39 kcal/mol). Hydrogen bonding provides a smaller but consistent stabilising effect (ΔG_Hbond_ = −1.37 to −2.24 kcal/mol). Although solvation penalties (ΔG_sol_ = 17.95 to 33.80 kcal/mol) partially offset these interactions, the overall binding remains strongly favourable. Ligand strain energies (2.24–8.58 kcal/mol) indicate some conformational adjustment upon binding, but this cost is outweighed by stabilising interactions. For PrKCα, the analogue shows a similarly favourable binding free energy (−37.82 kcal/mol), dominated by van der Waals (−35.59 kcal/mol) and Coulombic (−8.36 kcal/mol) terms, with moderate solvation penalties (21.87 kcal/mol) and low ligand strain (2.15 kcal/mol). Collectively, these results confirm that the analogue achieves stronger and more balanced interaction profiles with both MAPK1 and PrKCα compared with Brefeldin A, supporting its potential as a more stable, higher-affinity kinase-targeting candidate.

### 2.3. Cα-Root-Mean-Square-Deviation

**Figure 2:**
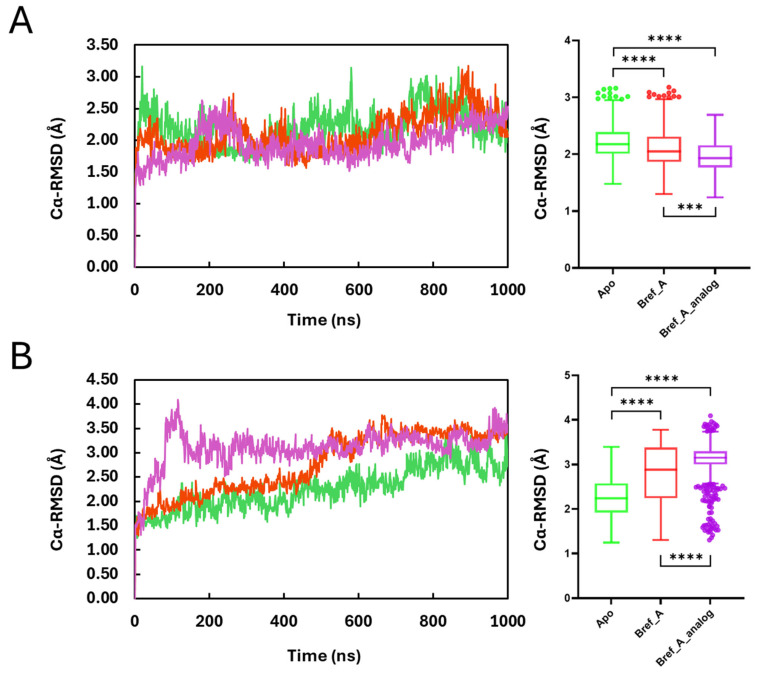
The root-mean-square deviation in the Cα atoms (Cα-RMSD) showing the evolution of MAPK1 (**A**) and PrKCα (**B**) on the left-hand panels, and the corresponding box and whisker plot summarising the distribution of the data. Green, red and magenta profiles represent Apo, Brefeldin A and Brefeldin A_analogue. The statistical analysis was performed using a non-parametric *t*-test based on the Mann–Whitney U test (centre line = median; box = IQR; whiskers = 1.5 × IQR; points = outliers; *** *p* < 0.001, **** *p* < 0.0001).

The conformational dynamics of the protein as apo or in complex with Brefeldin A and its analogue were measured by calculating the root-mean-square deviation in the alpha carbons (Cα-RMSD), which is a structural metric used to quantify how much a protein’s backbone conformation changes over time compared to a reference structure (often the initial or a crystal structure). Figure 2 shows how the protein evolved based on the rmsd of its Cα atoms, with respect to the 1000 ns simulation time. The results show that all three systems for both MAPK1 and PrKCα α (Apo, Brefeldin A complex and Brefeldin A analogue complex) converged within the 1000 ns simulation time. Overall, MAPK1_Apo is the least stable compared to the MAPK1:Brefeldin A and MAPK1:Brefeldin A analogue. The Box and Whisker plot and non-parametric *t*-test analysis show that the trajectories differ significantly from each other.

The MAPK1:Apo and MAPK1:Brefeldin A distributions differ significantly (*p* < 0.0001, two-tailed), with MAPK1:Apo showing a higher median RMSD (2.18 Å vs. 2.05 Å), corresponding to a modest reduction of about 0.13 Å (Hodges–Lehmann ~ −0.114 Å), and an effect size of Cliff’s δ ~ −0.22, meaning MAPK1:Brefeldin A frames have roughly a 61% chance of lower RMSD than MAPK1:Apo frames. For MAPK1:Apo vs. MAPK1:BrefA_analogue, the distributions also differ significantly (Mann–Whitney *p* < 0.0001, two-tailed), with MAPK1:Apo showing a higher median RMSD (2.18 Å vs. 1.93 Å), corresponding to a reduction of ~0.25 Å (HL shift ~ −0.24 Å) and an effect size of Cliff’s δ ~ −0.470 (medium-to-large), indicating ~73.5% probability that a random MAPK1:Brefeldin A-analogue frame has lower RMSD than MAPK1:Apo, thereby suggesting the Brefeldin A analogue stabilises and globally tightens the MAPK1 backbone more strongly than both in Apo and Brefeldin A. Hence, Brefeldin A binding is associated with a modest stabilisation of the backbone (slightly lower Cα-RMSD). The difference is statistically strong but small in magnitude, so it could be regarded as subtle stabilisation rather than a major conformational tightening.

The reverse is the case for PrKCα, which shows that the binding of the ligands significantly perturbs the conformation of the enzyme (Figure 2B). The PrKCα:Brefeldin A analogue showed the highest degree of deviation compared to PrKCα:Apo and PrKCα:BrefA. Statistically for PrKCα_Apo vs. PrKCα:BrefA complex, the distributions differ significantly (Mann–Whitney *p* < 0.0001, two-tailed), with PrKCα:Apo showing a lower median RMSD (2.24 Å) than PrKCα:Brefeldin A (2.88 Å), corresponding to an increase of ~0.64 Å (Hodges–Lehmann ~ 0.55 Å), indicating that Brefeldin A binding is associated with a measurable destabilisation and higher backbone deviation in PrKCα compared to the Apo state. For PrKCα Cα-RMSD: Apo vs. Brefeldin A_analogue (n = 1001 each), the distributions also differ significantly (Mann–Whitney *p* < 0.0001, two-tailed), with Apo-PrKCα showing a lower median RMSD (2.24 Å) than PrKCα:Brefeldin A_analogue complex (3.15 Å), corresponding to an increase of ~0.91 Å (Hodges–Lehmann ~ 0.89 Å), indicating that the Brefeldin analogue induces even greater backbone destabilisation of PrKCα than in Apo and by Brefeldin A.

**Figure 3:**
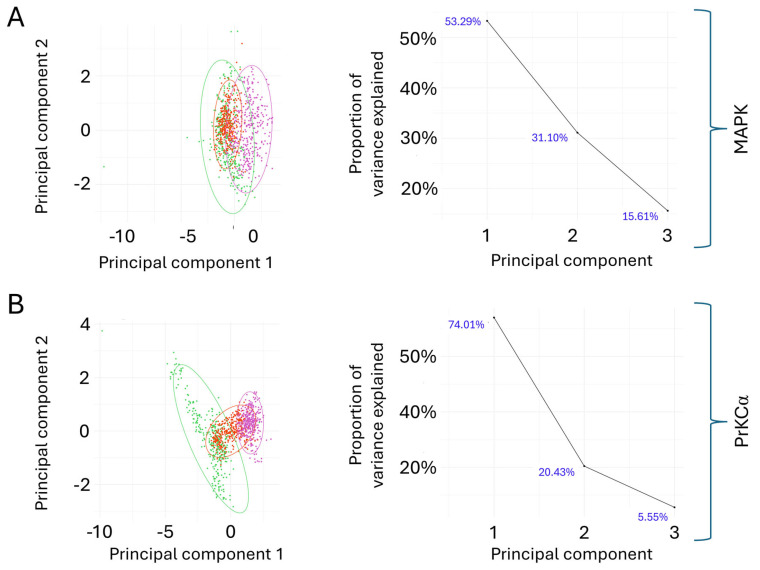
Principal Component Analysis (PCA) of the Cα-RMSD of MAPK1 (**A**) and PrKCα (**B**) across Apo, Brefeldin A-bound, and Brefeldin A_analogue-bound, illustrating clustering of the motion of the Cα atoms over the 1000-ns MD simulation time (green, red and purple dots represent Apo, Bref_A and Bref_A_analog, respectively).

The principal component analysis (PCA) of the Cα-RMSD trajectories (Figure 3) for MAPK1 revealed that the first two principal components captured approximately 84% of the total variance (PC1: 53.29%, PC2: 31.10%). The corresponding scree plot showed a gradual elbow after PC2, suggesting that two principal components were sufficient to describe the major conformational motions. In the PCA score plot, the apo form of MAPK1 exhibited the broadest dispersion, mainly distributed along negative values of PC1, indicating higher conformational heterogeneity. In contrast, the Brefeldin A-bound system was more tightly clustered and shifted toward the origin, suggesting a degree of stabilisation upon ligand binding. The Brefeldin A analogue further shifted toward positive PC1 values, occupying a conformational space partially distinct from both the apo and Brefeldin A complexes. These findings suggest that ligand binding induces moderate reorganisation of the conformational ensemble in MAPK1, with overlapping but distinguishable shifts between apo and liganded states.

For PrKCα, PCA revealed a more pronounced effect of ligand binding. The first principal component alone accounted for 74.01% of the total variance, with PC2 accounting for a further 20.43%, indicating that a single dominant motion described most conformational changes. The scree plot displayed a sharp elbow after PC1, consistent with this observation. In the PCA score plot, the apo form of PrKC again displayed the widest distribution, clustered at negative PC1 values, consistent with greater conformational flexibility. By contrast, both the Brefeldin A- and analogue-bound systems shifted toward higher PC1 values and exhibited more compact clusters, indicative of reduced heterogeneity and enhanced structural stabilisation. Although Brefeldin A and its analogue overlapped substantially, subtle differences in their clustering patterns suggested non-identical conformational preferences.

### 2.4. Radius of Gyration

**Figure 4:**
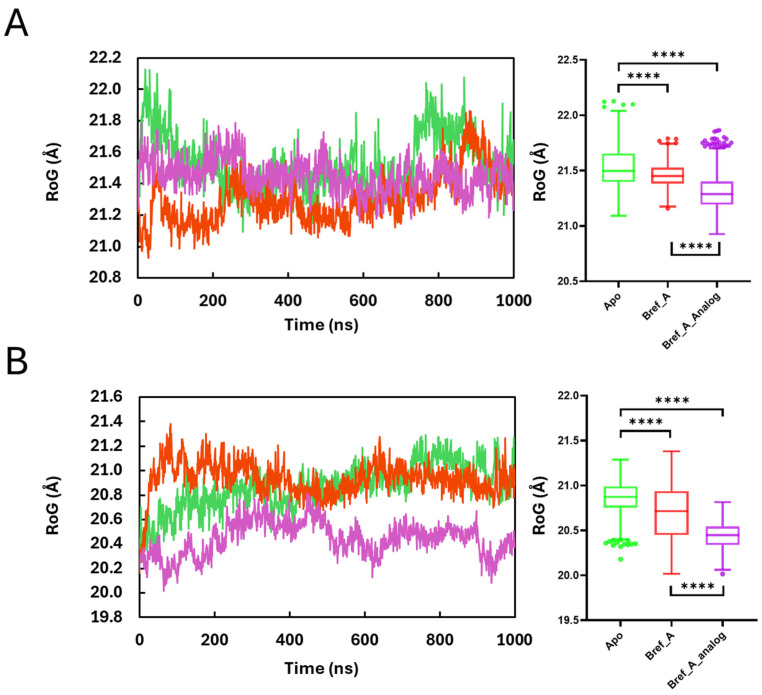
Time evolution and distribution of protein compactness (RoG) for (**A**) MAPK1 and (**B**) PrKCα. Left: time traces show the Cα radius of gyration (RoG) over 1000 ns MD for Apo, Brefeldin A, and Brefeldin A analogue; trajectories were fitted on protein Cα atoms, and RoG was computed at a 1 ns stride. Right: box-and-whisker plots summarise the same data (centre line = median; box = IQR; whiskers = 1.5 × IQR; points = outliers; **** *p* < 0.0001).

The radius of gyration (RoG) is a structural descriptor that measures how compact or extended a molecule is during a simulation. Figure 4 shows that the binding of Brefeldin A and its analogue impacts the compactness of MAPK1 in comparison with the MAPK1:Apo enzyme. For MAPK1 (MAPK1:Apo vs. MAPK1:Brefeldin A; n = 1001 each), the distributions differ significantly (Mann–Whitney *p* < 0.0001, two-tailed), with Apo showing a slightly higher median (21.50 Å) than Brefeldin A (21.45 Å), a shift of approximately −0.05 Å (HL ~ −0.057) and a small effect (Cliff’s δ ~ −0.222; ~61% chance a frame from MAPK1:Brefeldin A complex has lower RoG), indicating only subtle compaction upon ligand binding. Concerning Apo vs. Brefeldin A_Analogue, the distributions differ significantly (Mann–Whitney *p* < 0.0001, two-tailed), with Apo showing a higher median (21.50 Å) than the analogue (21.29 Å), which is a shift of approximately −0.21 Å (Hodges–Lehmann ~ −0.218) and a large effect (Cliff’s δ ~ −0.659; ~83% chance a frame from MAPK1:Brefeldin A analogue is more compact), indicating marked global compaction upon analogue binding, compared to Brefeldin A binding.

Similarly, for the PrKCα systems, the overall results show that binding of Brefeldin A or its analogue increases the compactness of the enzyme. For the Apo PrKCα system vs. PrKCα:Bref A complex, (n = 1001), the distributions differ significantly (Mann–Whitney *p* < 0.0001, two-tailed), with medians 20.87 Å (Apo) vs. 20.71 Å (Brefeldin A) giving a HL shift −0.166 Å and Cliff’s δ ~ −0.331 (~66.5% chance a PrKCα:Brefeldin A frame is more compact than an Apo frame), indicating moderate global compaction upon ligand binding. Also, for PrKCα_Apo vs. PrKCα:Brefeldin A_analogue complex (n = 1001 each), the distributions differ significantly (Mann–Whitney *p* < 0.0001, two-tailed), with a lower median RoG for the analogue (20.45 Å) than Apo (20.87 Å), a shift of ~−0.43 Å (HL ~ −0.427), and a very large effect (Cliff’s δ ~ −0.917; ~96% chance an analogue frame is more compact), indicating definite global compaction upon Brefeldin A analogue binding.

### 2.5. Cα Root-Mean-Square Fluctuation

**Figure 5:**
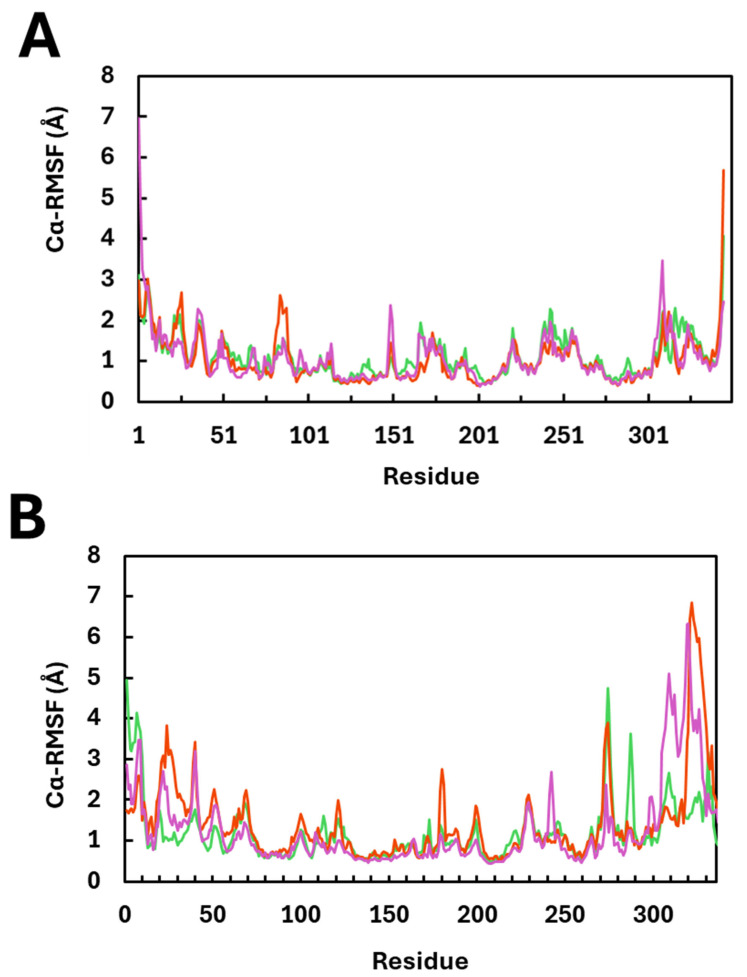
Per-residue Cα RMSF for apo- and ligand-bound kinases. (**A**) MAPK1: Apo, Brefeldin A, Brefeldin A_analogue. (**B**) PrKCα: Apo, Brefeldin A, Brefeldin A_analogue. RMSF (Å) was calculated for each residue after superposing all frames on the protein Cα backbone of a reference structure to remove rigid-body motion; trajectories span 1 µs and were sampled at ~1 ns stride (green, red and purple lines represent Apo, Bref_A and Bref_A_analog, respectively).

Cα root-mean-square fluctuation (Cα-RMSF), which is a measure of the average positional deviation in an atom or group of atoms over time, relative to its average position in a molecular dynamics trajectory. When calculated for Cα atoms specifically, it reflects the fluctuations of the backbone alpha carbons of amino acids, which form the protein’s structural framework. A one-way ANOVA was performed to determine the effect of Brefeldin A binding on the Cα-RMSF, using a non-parametric Kruskal–Wallis analysis. Figure 5 summarises the RMSF values obtained for the MAPK1 and PrKCα enzymes following a 1000 ns MD simulation in the presence or absence of Brefeldin A or its analogue using the Desmond engine. In Figure 5, it can be generally observed that the binding of Brefeldin A or its analogue to MAPK1 did not impact the native fluctuations of the Cα atoms during the simulation. However, we cannot discount the C-terminus region of MAPK1, where moderate fluctuations were observed. The PrKCα system showed more pronounced differences in fluctuations of the Cα atoms due to Brefeldin interaction. Figure 5B shows that the N-terminus region of the PrKCα varied significantly in fluctuation (Kruskal–Wallis H(2) = 21.44H, N = 150, *p* < 0.0001) across the three systems. The RMSF was more pronounced in the last 37 residues of the enzyme (Kruskal–Wallis H(2) = 18.15, N = 111, *p* < 0.0001), showing that Brefeldin A interaction with PrKCα resulted in more fluctuations about their average position during the simulation.

**Figure 6:**
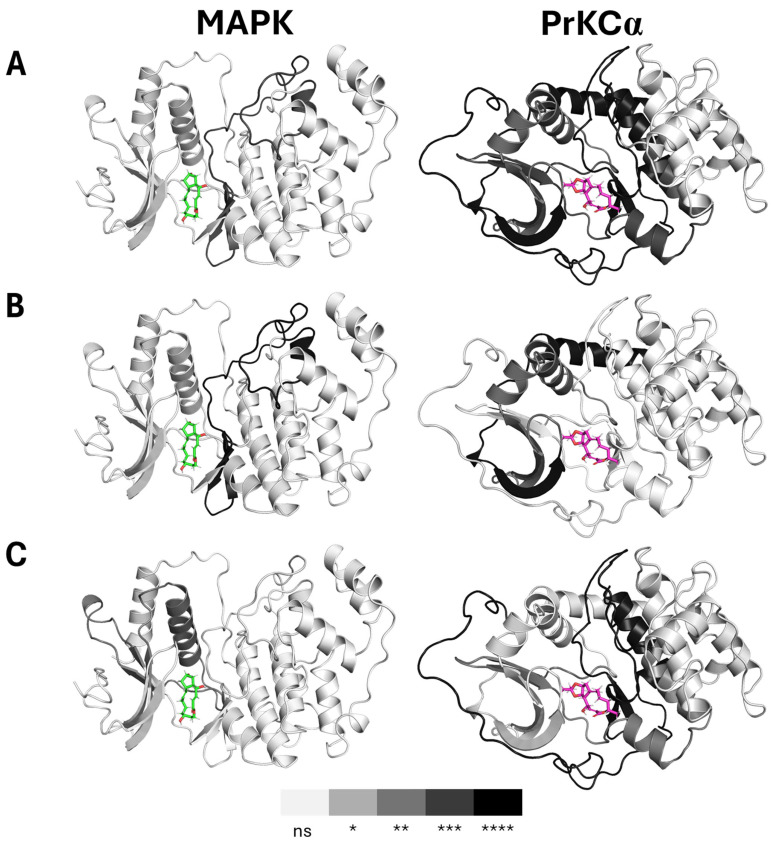
Variation in Cα-RMSF profiles of MAPK1 and PrKCα under different ligand-binding conditions. (**A**) Non-parametric one-way ANOVA showing overall variation across Apo, Brefeldin A, and Brefeldin A analogue-bound sub-groups. (**B**) Non-parametric pairwise comparison (Mann–Whitney U test) between Apo and Brefeldin A-bound enzymes. (**C**) Non-parametric pairwise comparison between Apo and Brefeldin A analogue-bound enzymes. The structural representations illustrate residue-level fluctuations, with darker shading highlighting regions with greater significance. The colour bar denotes the statistical significance levels: ns = non-significant, * *p* < 0.05, ** *p* < 0.01, *** *p <* 0.001, and **** *p* < 0.0001. The stick structure is bound Brefeldin A.

In Figure 6, we applied both a non-parametric one-way ANOVA and pairwise *t*-tests to examine the degree of variation in 50-residue segments of the protein across the Apo-protein, protein:Brefeldin A (Brefeldin A), and protein:Brefeldin A analogue (Brefeldin A_analogue) complexes. These analyses allowed us to identify regions where fluctuations in the Cα-RMSF were either insignificant or statistically significant. In the MAPK1 systems, the most pronounced variation (*p* ~ 0.001) occurred within residues 150–200, encompassing β7–β9 and α7–α8, which form part of the active site. Most other regions did not display significant variation (*p* > 0.05). Pairwise comparisons confirmed this trend: the Apo vs. Brefeldin A systems showed the strongest differences in residues 150–200, while the Apo vs. Brefeldin A_analogue systems exhibited the greatest variation in residues 51–100 (β3–α3–α4–β4) with *p* < 0.01. These findings suggest that Brefeldin A binding does not induce substantial fluctuations in the backbone dynamics of MAPK1.

In contrast, the PrKCα systems displayed considerably greater variation in backbone fluctuations upon Brefeldin A interaction. Significant differences (*p* ~ 0.001 to *p* < 0.0001) were observed in residues 1–50 (β1–β3), 101–150 (α2–α3–β7–α4–β8), 151–200 (β9–β10–α5–α8), and 300–337 (α15–α17). Pairwise analyses revealed only minor differences between Apo and Brefeldin A, with the most notable variation occurring at residues 1–50 (*p* < 0.0001). By contrast, Apo vs. Brefeldin A_analogue comparisons showed consistently high variation, particularly within residues 151–200 and 300–337 (*p* < 0.0001).

### 2.6. Brefeldin A Binding Properties

Ligand RMSD (with respect to the receptor) is computed by first aligning each trajectory frame to the protein, typically the Cα/backbone or selected pocket residues, and then calculating the RMSD of the ligand’s heavy atoms to a reference pose. This removes the protein’s overall rotation and translation, so the value reflects how much the bound pose shifts or deforms relative to the receptor over time. Low, plateaued ligand RMSD indicates a stable binding pose; large or sustained increases suggest reorientation, weak binding, or partial dissociation.

**Figure 7:**
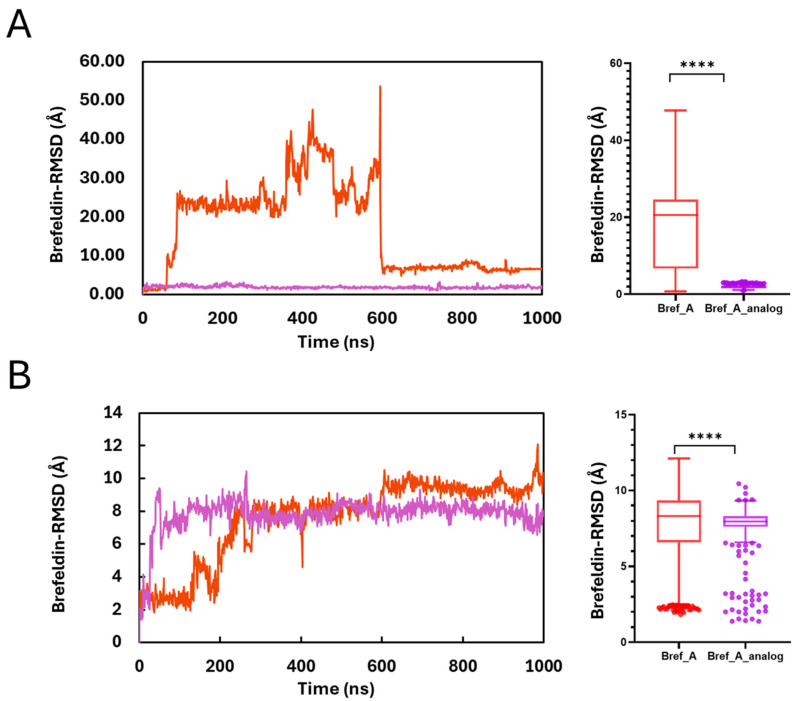
Ligand pose stability from ligand-RMSD relative to the receptor. (**A**) MAPK1 and (**B**) PrKC complexes with Brefeldin A or Brefeldin A_analogue. For each trajectory, frames were aligned to the receptor Cα backbone and the RMSD of the ligand heavy atoms to the reference pose was computed (Å) at ~1 ns stride over 1000 ns. Left panels show time evolution; right panels summarise the same data with box-and-whisker plots (centre line = median, box = IQR, whiskers = 1.5 × IQR, points = outliers, **** *p* < 0.0001, red and purple profiles represent Bref_A and Bref_A_analog, respectively).

**Figure 8:**
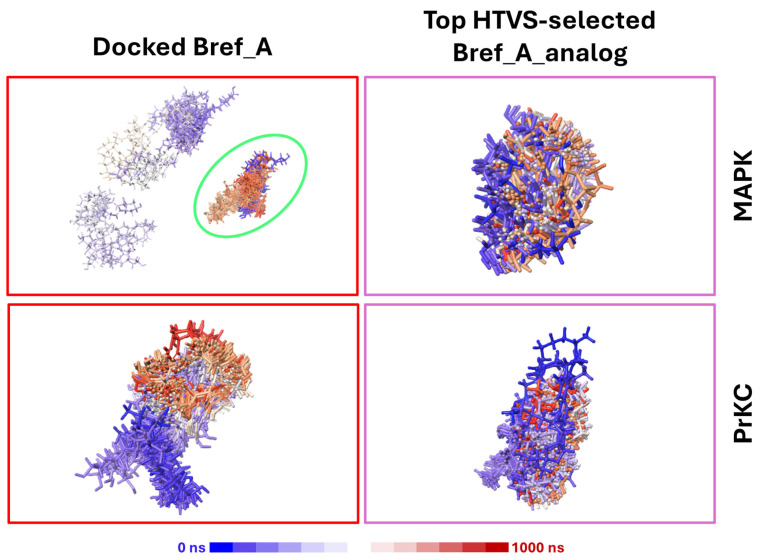
Time-coloured overlays of ligand positions during 1000 ns MD simulation. Superposed ligand coordinates are shown as sticks coloured by simulation time from 0 ns (blue) to 1000 ns (red) after aligning each frame to the receptor binding site (protein omitted for clarity). The green ellipse highlights the dominant pose MAPK1:Brefeldin A among other poses. The colour bar denotes the time gradient.

Figure 7A shows the RMSD of Brefeldin A/Brefeldin A analogue with respect to MAPK1. The results indicate that Brefeldin A binding was not stable enough to keep the compound bound to MAPK1. This is because of an enormous RMSD (Median ~ 21 Å). The trajectory shows that Brefeldin A left the binding pocket at the 60th ns and came back to the same binding pocket at the 600th ns. In comparison, the Brefeldin A analogue remained stably bound to the receptor throughout the 1000 ns simulation (Median ~ 1.89 Å). Figure 7B shows that Brefeldin A and its analogue remained bound to the PrKCα receptor throughout the 1000 ns simulation time. Thus, making PrKCα a more preferred receptor for Brefeldin A compared to MAPK1. Nonetheless, the Brefeldin A analogue appears to bind more stably in PrKCα than to Brefeldin A. Figure 8 correlates the Lig-RMSD (Figure 7), showing the positions of the ligand every 10 ns over the 1000 ns simulation time. Top LHP shows how Brefeldin A wandered off the initial binding pocket and returned to it. This is exemplified by the cohort of Brefeldin A circled in green. A closer examination of that cohort of ligands shows ligands coloured blue and red, depicting ligands at the first few ns of the simulation and at later times in the trajectory. This is unlike the ligands in the top RHP and bottom panels, which remained clustered together throughout the 1000 ns MD simulation.

**Figure 9:**
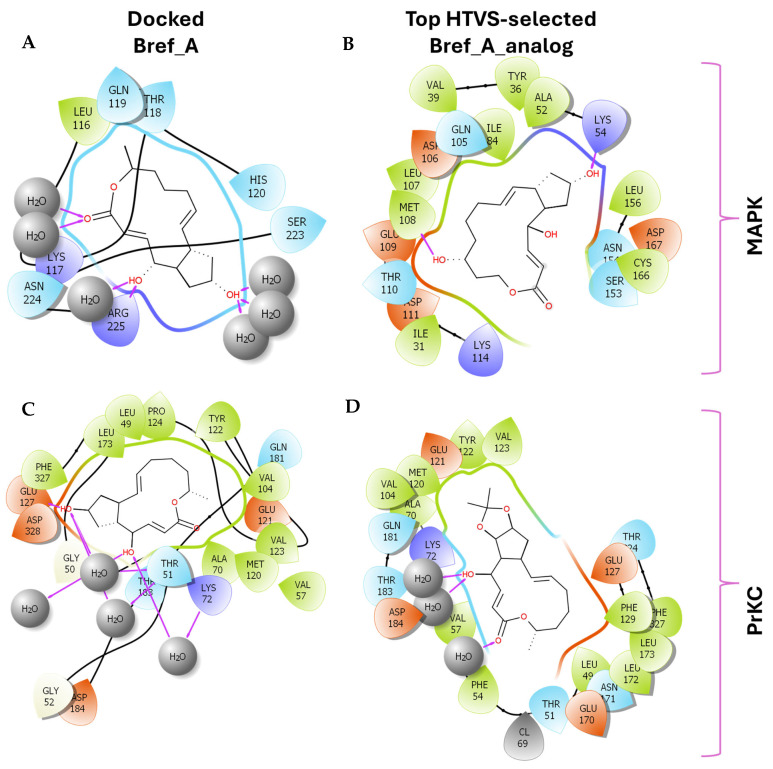
Two-dimensional interaction diagrams of Brefeldin A and its analogue bound to MAPK1 and PrKCα active sites. The figure shows ligand–protein interactions as determined from molecular dynamics simulations. Residues surrounding the ligands are displayed as coloured circles, with colour coding based on residue type (hydrophilic, hydrophobic, polar, charged). Hydrogen bonds are indicated with green dashed lines, hydrophobic contacts with orange arcs, and water-mediated interactions with grey spheres labelled H_2_O. Ligand hydroxyl groups are marked in red, while halogen or ionic interactions are indicated where relevant. (**A**) MAPK1:Brefeldin A, (**B**) MAPK1:Brefeldin A analogue, (**C**) PrKCα:Brefeldin A, and (**D**) PrKCα:Brefeldin A analogue.

Figure 9 illustrates a simplified 2D orientation of the interaction between Brefeldin A/and its analogue with the protein kinases. The results show key residues that stabilise the ligand within the active site. The pose was generated from the trajectory clustering analysis implemented in Meastro v13. Basically, what this module does is cluster groups of MD frames into conformational states by aligning structures, computing RMSD over selected atoms, and applying RMSD-based clustering to partition snapshots and report cluster populations. It identifies protein or ligand poses, compares apo/holo landscapes, and selects representative structures for MM/GBSA. The figure supports the low interaction potential between Brefeldin A and MAPK1 and supports the tighter binding of Brefeldin A/analogue to PrKCα.

**Figure 10:**
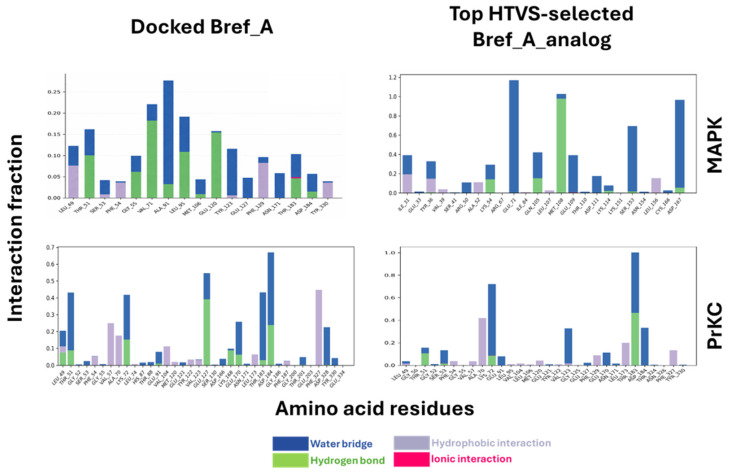
Interaction frequency histograms for Brefeldin A and its analogue bound to MAPK1 and PrKCα during molecular dynamics simulations. The plots show the relative frequency of protein–ligand interactions over 1000 ns simulations. Interaction types are colour-coded as shown below the figure. Key residues mediating stable contacts are highlighted, indicating differences in binding stability and interaction profiles between Brefeldin A and its analogue across the two receptors.

Besides H-bonding interaction, there are other forces of interaction between Brefeldin A and the residues in the binding site. Figure 10 is a stacked bar plot of the various interactions that stabilised Brefeldin A and the active site. This includes water bridges, ionic interaction, van der Waals contact, etc. This figure summarises the fraction of interaction between Brefeldin A/Brefeldin A analogue with the residues within the binding pocket. The higher the number of contacts and the lower the fraction of interaction, the more unstable the binding, potentially leading to the ligand exiting the binding pocket, and vice versa. Figure 8 corroborates the weak binding of Brefeldin A to MAPK1, as there is a dramatic increase in the number of amino acid residues making contact with the ligand. On the other hand, the other panels have a higher fraction of interaction and fewer amino acid residues stabilising the ligands within the binding pocket.

**Figure 11:**
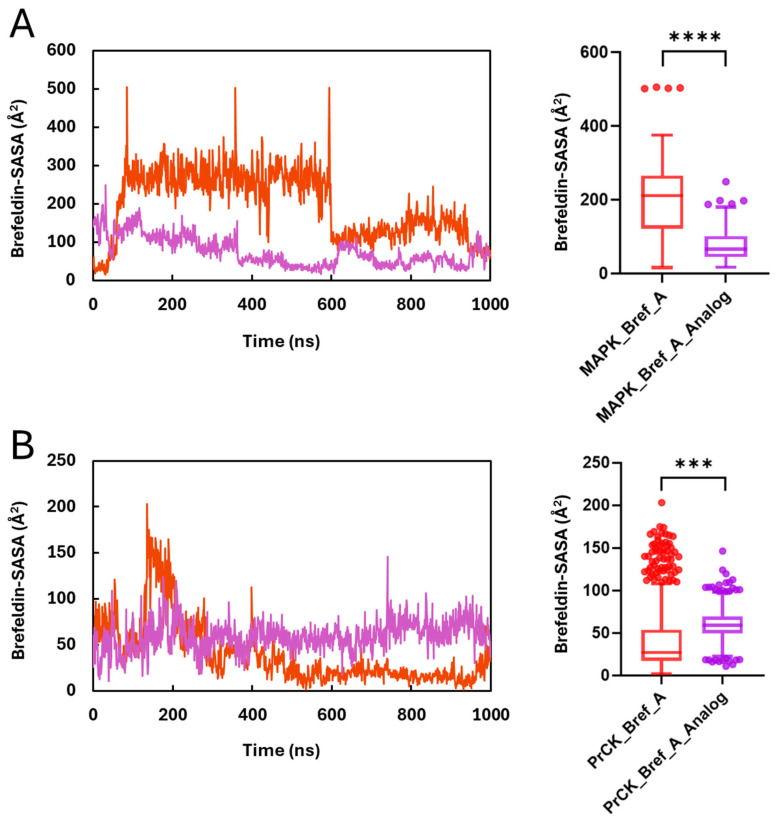
Ligand solvent exposure during MD, quantified by SASA. (**A**) MAPK1 complexes and (**B**) PrKC complexes with Brefeldin A or Brefeldin A_analogue. For each frame of the 1 µs trajectories, the ligand solvent-accessible surface area (SASA) was computed for heavy atoms with a 1.4-Å probe (Shrake–Rupley) at ~1 ns stride. Left panels show SASA time series; right panels summarise the same data with box-and-whisker plots (centre line = median, box = IQR, whiskers = 1.5 × IQR, points = outliers, *** *p* < 0.001, **** *p* < 0.0001).

Finally, ligand SASA (solvent-accessible surface area) is the area of the ligand’s surface that a solvent probe (usually a 1.4 Å water-sized sphere) can “touch,” which is computed per frame while the ligand is in complex with the protein. These metrics indicate ligand stability and exposure of the ligand to solvent molecules while it is bound to the receptor. Figure 11 illustrates the SASA of Brefeldin A and its analogue, while bound to the two protein kinases. The MAPK1 result corroborates the ligand RMSD, indicating that Brefeldin A dissociates from the binding pocket within the 60 ns and re-associates with the binding site from the 600th ns. It also shows that the analogue of Brefeldin A is more stable in MAPK1 than Brefeldin A. Furthermore, the figure shows that the analogues of Brefeldin A form a more stable interaction, as indicated by the lower SASA values compared with Brefeldin A for both protein kinases. The variation between the SASA trajectories is significant (*p* < 0.0001) using the non-parametric *t*-test.

### 2.7. MD-Simulation-Based MM/GBSA Free Binding Energy Calculation

The MM/GBSA analysis (Table 4) of trajectories from the 500 ns simulation shows that binding affinity (Δ*G*_bind_) is strongest for MAPK1_Brefeldin A (−33.64 ± 5.54 kcal/mol), followed by MAPK1_Brefeldin analogue (−28.83 ± 3.97), PrKCα_Brefeldin A (−24.05 ± 7.52), and PrKCα_Brefeldin analogue (−19.86 ± 3.00), indicating a consistent preference of both ligands for MAPK1 over PrKCα by about 9–10 kcal/mol and a stronger performance of Brefeldin A compared to its analogue within each protein (~4–5 kcal/mol advantage). The gas-phase contribution (Δ*G*_gas_), which combines van der Waals and electrostatic terms, is most favourable for MAPK1_Brefeldin A (−56.56 ± 10.26), driven by strong van der Waals interactions (−37.31 ± 3.92) and favourable electrostatics (−19.24 ± 9.24). In general, MAPK1 complexes exhibit more favourable gas-phase energies than PKCα complexes, which aligns with their overall stronger binding free energies. All complexes incur a positive solvation penalty (Δ*G*_sol_), partially offsetting the gas-phase stabilisation, with the largest penalty observed in PKCα_Brefeldin Analogue (26.45 ± 6.83), contributing to its weakest binding affinity. Notably, electrostatic and gas-phase terms show higher variability, particularly in PKCα_Brefeldin A (Δ*E*_ele_ SD 12.59; Δ*G*_gas_ SD ± 15.57), reflecting greater conformational heterogeneity during the simulation. Overall, binding in these systems is primarily driven by van der Waals interactions supported by electrostatics. At the same time, solvation counteracts binding, and the MAPK1 binding pocket emerges as a more favourable environment for both ligands, especially Brefeldin A.

**Figure 12:**
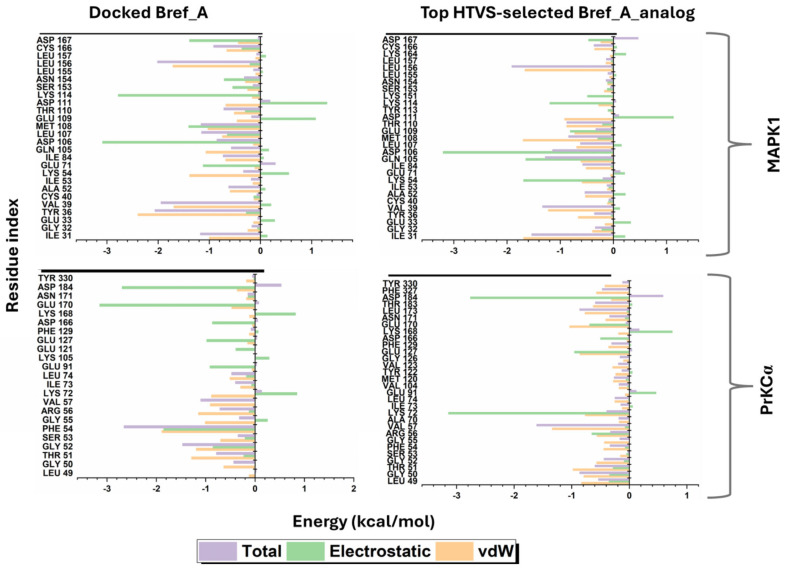
Per-residue free energy decomposition of protein–ligand binding calculated using the MM/GBSA method from snapshots extracted over a 500 ns molecular dynamics trajectory. Residues contributing to ligand binding are plotted with their respective energy components: van der Waals (vdW, orange), electrostatic (green), and total binding free energy contribution (purple). Negative values indicate favourable contributions to ligand binding. The upper panels correspond to MAPK1–ligand complexes (Brefeldin analogue, (**left**); Brefeldin A, (**right**)), while the lower panels correspond to PrKCα–ligand complexes (Brefeldin analogue, (**left**); Brefeldin A, (**right**)). Error bars represent the standard error of the mean from trajectory sampling.

Information from the per-residue plots indicated important active site residues contributing electrostatic energy to the binding of Brefeldin and its analogue to the two pockets (Figure 12). Additionally, Glu 109 and Lys 54, which favourably contributed electrostatically to binding, showed an unfavourable electrostatic energy contribution to MAPK1 binding. At the same time, Asp 111 in both MAPK1_Brefeldin analogue and MAPK1_Brefeldin A gave an unfavourable electrostatic energy contribution to binding. Moving forward, to investigate the per-residue energy contributions to Brefeldin and its analogue binding to PrKCα, electrostatic energy also proved to be quite prominent, differing in Lys 72, which contributed unfavourably to the electrostatic energy in the binding of Brefeldin analogue to PrKCα, and favourably in the binding of Brefeldin to PKCα. In contrast, Lys 168 exhibited an unfavourable electrostatic energy contribution to both MAPK1 and PrKCα. These are in addition to other residues that also contribute to the electrostatic and van der Waals energies of Brefeldin and its analogue binding to MAPK1 and PKCα.

## 3. Discussion

A study by Liu et al. [37] found a connection between glycosylation and the Ras/Raf/MAPK1 signalling pathway in carcinoma (HCC) cells treated with sorafenib, a multi-kinase inhibitor. Treatment of HCC cells with sorafenib modified protein glycosylation. Additionally, changes were observed in how glycoproteins bind to lectins following sorafenib treatment. These alterations in glycosylation profiles indicated that sorafenib impacted the Ras/Raf/MAPK1 signalling pathways [37]. By inhibiting these pathways in HCC cells, sorafenib decreased the expression of Ets1 (erythroblastosis virus E26) associated with protein glycosylation. The suppression of Ets1 expression after sorafenib treatment suggests a relationship between this signalling pathway and glycosylation processes within HCC cells [37]. Furthermore, the study proposes that influencing the sorafenib’s Ras/Raf/MAPK1 signalling pathway indirectly affects protein glycosylation patterns in HCC cells, potentially impacting cancer progression. Their research highlighted the interaction between signal transduction pathways and post-translational modifications, such as glycosylation in cancer cells [37].

The identification of probable protein targets for Brefeldin A using Swiss Protein Predict yielded numerous probable targets. By combining the Swiss Protein Predict results with further computational and experimental analyses, we can gain valuable insights into the molecular mechanisms underlying Brefeldin A’s effects on cancer cells. This knowledge will ultimately contribute to developing more effective therapeutic strategies targeting colorectal cancer and potentially other types of cancer. This led to the identification of two proteins of interest.

Protein kinase C (PKC) is a family of serine/threonine kinases that regulates proliferation, differentiation, survival and apoptosis, placing it at pivotal control points in oncogenesis [38,39,40]. Isoform effects are context-dependent: PKCα can drive growth signalling in breast cancer (e.g., JAK/STAT, PI3K/AKT) [39]; PKCη shows tumour-promoting or suppressive roles depending on tissue [41,42]; PKCε promotes survival, epithelial–mesenchymal transition (EMT), invasion and metabolic reprogramming [43,44]; and PKCδ can support either cell death or survival depending on cellular context [40,45]. PKC also regulates glycosylation, a modification that shapes receptor stability, trafficking, adhesion and immune recognition in cancer [6,46,47]. PKC inhibitors (e.g., staurosporine) can remodel *N*-glycan biosynthesis by lowering *N*-acetylglucosaminyltransferase V activity, reducing β1,6-branching, strengthening tumour-cell adhesion to the extracellular matrix and suppressing metastatic potential [48]. More broadly, PKC tunes glycosyltransferase activity, enzyme localisation and membrane trafficking, thereby rewiring the glycosylation of receptors and adhesion molecules and altering downstream signalling and cell–matrix interactions that drive progression [49].

In another study focused on neuroblastoma (NB) cells, researchers examined the role of phosphoinositide 3-kinase (PI3K) in glycosylation and the distribution of molecules affecting NB cell properties. The study explicitly highlights how changes in NB cell properties are influenced by the status of glycosylation and molecular localisation via the PI3K/PKC pathways. It demonstrates that both PI3K and PKC play a role in regulating glycosylation processes that impact cell behaviour in neuroblastoma [50].

The role of MAPKs in glycosylation has been studied in the yeast Saccharomyces cerevisiae. The focus was on the high osmolarity glycerol (HOG) and filamentous growth (FG) pathways. These pathways employ MAPKs, such as Hog1 in the HOG pathway and Kss1 in the FG pathway. Each MAPK1 responds to stimuli and plays distinct roles in the yeast’s response to environmental conditions. During the study, researchers discovered two mutants, mnn10 and mnn11, that activated Kss1, an important MAPK1 involved in the FG pathway. These mutants lacked the MNN10 and MNN11 genes, which encode mannosyltransferase enzymes, critical for the Golgi apparatus and the *N*-glycosylation machinery. Deleting these genes resulted in the production of *N*-glycosylated proteins with shorter mannan chains. The findings highlight the significance of *N*-glycosylation in maintaining accurate MAPK1 signalling, particularly by preventing cross-activation between different MAPK1 pathways. Additionally, researchers identified an *N*-glycosylation site on Msb2 (Asn 30), which, when mutated, led to the activation of Kss1, similar to what was observed in the mnn11 mutant phenotype. This finding also highlights the significance of *N*-glycosylation in regulating MAPK1 signalling for Msb2, a crucial regulator, in the FG pathway [13].

In a typical study of this kind, molecular docking is the first computational modelling step following protein preparation. Docking provides initial insights into how a ligand might be recognised by a protein receptor. In our work, we employed AutoDock Vina [51] to predict how Brefeldin A (PubChem CID: 5287620) might bind within the active sites of MAPK1 and Protein Kinase C alpha (PrKCα). This is because the Induced Fit Docking tool implemented in Maestro does not dock macrocyclic compounds, such as Brefeldin. After obtaining plausible binding poses, we performed a 250-nanosecond molecular dynamics (MD) simulation to test the stability of the Brefeldin A:MAPK1 complex. These MD simulations revealed that Brefeldin A does not maintain stable interactions with MAPK1 over time. Because of this instability, we turned to the PubChem database to search for Brefeldin A analogues, with the idea that some of them might form more stable interactions. Notably, target prediction analyses suggested that MAPK1 remains a likely protein target for these analogues, in addition to the parent compound. Using a library of Brefeldin A and its analogues, we identified two analogues that showed significantly better preference in docking and binding for either MAPK1 or PrKCα. Ee used Prime MM/GBSA (part of the Schrödinger Maestro v13 suite) to compute binding free energies to validate these findings further [52]. These calculations indicated that the analogues bound to the receptors spontaneously and more favourably than the parent compound in the simulated environment.

Building on these docking and free-energy analyses, we next examined the trajectory patterns and dynamic behaviour of Brefeldin A and its analogues during MD simulations to assess their binding stability and inhibitory potential further. The trajectory pattern obtained for the binding of Brefeldin A to these two proteins showed that Brefeldin A binding, especially its analogues, produced a trajectory pattern, showing inhibitory activity. An investigation into the binding pattern of Brefeldin A when docked into MAPK1 and PrKCα showed that Brefeldin A binds in different pockets. This may be attributed to Brefeldin A’s relatively weak binding affinity; hence, instability at one pocket results in binding to different pockets. Additional studies could identify events that might produce inconsistent pocket binding.

The trajectory pattern and the average RMSF suggest that binding Brefeldin A did not induce significant structural changes in residue motions relative to the MAPK1_apo form. Given the minimal fluctuations observed, this residue pattern suggests greater rigidity relative to the apo form. In this regard, previous studies have associated rigidity with inactivity [53]. Hence, from the observations made from the Cα-RMSD and Cα-RMSF plots, it can be inferred that the binding of Brefeldin A induced a structural alteration, which could presumably produce inhibition of MAPK1 and PrKCα. However, additional studies could substantiate this view. The mobility pattern obtained for Brefeldin A-bound MAPK1 did not show a distinct mobility difference, though Brefeldin A-bound MAPK1 exhibited a RoG pattern higher than that of the apo. In the case of Brefeldin A-bound PrKCα, there was a mobility difference between Brefeldin A-bound PrKCα and the apo form, which indicates the inactivity of PrKCα. Overall, PCA affirmed that ligand binding has a stabilising effect on both proteins, but the magnitude of this effect differed markedly between MAPK1 and PrKC. While MAPK1 exhibited partial separation of apo and liganded states with moderate conformational reweighting, PrKC showed a stronger ligand-induced stabilisation largely captured along a single conformational mode. These differences underscore the protein-specific nature of ligand-induced conformational dynamics.

The trajectory of the ligand RMSD plot indicated that Brefeldin A is bound with a stable and consistent configurational dynamic. Throughout the MD simulation, the 3D presentation of the orientation of Brefeldin A’s configuration during the 1000 ns MD simulation showed that the structural packing of Brefeldin A and its conformational configuration remained similar in both MAPK1 and PrKCα (Figure 5). As reported here, the two trajectories for binding Brefeldin A to MAPK1 and PrKCα were quite similar but with high Å^2^, suggesting that Brefeldin A established a sufficiently strong level of interaction, which will produce binding that could result in functional activity/inactivity. The sudden trajectory jump observed in the SASA plot could be due to the conformational reorientation of Brefeldin A at that binding pocket of PrKCα. The consistent horizontal trajectory of the plot pictures suggests that Brefeldin A remained in the binding pockets of MAPK1 and PrKCα and was not displaced. The comparative 2D interaction diagram revealed that the presence of bulk water in the MD simulation medium did not appear to contribute much to the dynamics and binding stabilisation of Brefeldin A- MAPK1/PrKCα complexes, as there was insufficient water to contribute to hydrogen bonds to stabilise Brefeldin A. The binding and interactions might be produced by other factors which promote molecular recognition and subsequent binding.

The nature and types of interactions established between Brefeldin A and MAPK1 and PrKCα could provide further insight into the silent structural events that might have produced the obtained binding stabilisation of Brefeldin A on the pockets of the two proteins. Figure 8 shows that the scarcity of bulk water led to reduced hydrogen bonding between Brefeldin A and MAPK1, while promoting a higher incidence of water bridges. Hydrogen bonding is more prevalent in the binding of Brefeldin A to PrKCα. As shown in Figure 9, the active site residues that played key roles in stabilising the binding of Brefeldin A are presented, along with their corresponding percentage occupancies.

It is essential to highlight the role of bulk water observed in both average PDBs, where it contributes equally to binding stabilisation. This observation reinforces the conclusion presented in Figure 7 regarding the critical role of water in ligand binding. Furthermore, the stacked bar charts (Figure 10) show that water bridges, van der Waals forces, and hydrogen-bonding interactions are the dominant contributors to ligand–residue contacts within the active site. Interestingly, although a greater number of residues in PrKCα contributed to the stabilisation of Brefeldin A, the overall energy outputs for both proteins remained comparable.

We also observed a notable disparity between the Amber-18-based MMGBSA calculations and the results obtained using HTVS/Prime MMGBSA and molecular dynamics (MD) simulations. Initially, we expected the Amber-18-based MMGBSA values to be consistent with those generated using Schrödinger Maestro algorithms, given that both approaches rely on similar principles. However, the differences can be explained by the distinct factors and scoring schemes used in docking and MMGBSA calculations.

Overall, disparities between binding free energies predicted by MMGBSA and those obtained from docking or molecular dynamics (MD) simulations reflect fundamental differences in their assumptions, accuracy, and computational treatment of binding. Docking algorithms, widely used for ligand ranking, employ simplified scoring functions that emphasise van der Waals and electrostatics while neglecting solvation and entropy. This often results in underestimated affinities, particularly for flexible or charged ligands [54,55,56]. MMGBSA provides a more refined estimate by incorporating solvation and entropic contributions, but it can overestimate binding due to limitations in treating long-range electrostatics and charged molecules [57]. MD-based methods add realism by sampling conformational flexibility, yet their outcomes depend strongly on trajectory length, equilibration, and initial conditions. Inadequate sampling can bias the ensembles used for MMGBSA, while docking is more dependent on initial binding poses [58,59]. Nonadditive protein–ligand interactions pose additional challenges: docking fails to capture these effects, MMGBSA approximates them, and only alchemical free-energy methods provide rigorous treatment at high computational cost [54,60]. Finally, MMGBSA depends on the choice of force field and typically uses an implicit solvent, which misses local water effects captured in explicit solvent MD [61,62].

Generally, the findings of this study are consistent with current research on the effects of Brefeldin A on protein structure. Studies have demonstrated that Brefeldin A, an inhibitor of N-linked glycosylation and protein trafficking between the endoplasmic reticulum (ER) and Golgi apparatus, can induce ER stress and disrupt Golgi structure, ultimately affecting protein folding and stability [63]. The observed conformational changes and increased flexibility in both PrKCαa and MAPK1 suggest that Brefeldin A interferes with their structural integrity, potentially leading to altered protein function. Variations in compactness further indicate that Brefeldin A affects the overall folding and shape of these proteins, with significant implications for their biological activities [64].

## 4. Materials and Methods

### 4.1. Identification of Target Proteins

The Chemical Entities of Biological Interest (ChEBI) database (https://www.ebi.ac.uk/chebi/, accessed on 14 February 2023) was first used to obtain the spatial data file (.sdf) and the SMILES structure of Brefeldin A. The SMILES files for the Brefeldin A ligands were analysed using SwissTargetPrediction (https://www.swisstargetprediction.ch/, accessed on 2 July 2025) to generate a ranked list of predicted protein interactors. Docking scores from both platforms were used to prioritise targets, and a stringency factor of eight was applied to retain only high-confidence candidates. A stringency factor of eight was selected because it represents a high-confidence threshold commonly used in proteomic and network-based filtering pipelines. This value balances sensitivity and specificity by minimising false positives while retaining biologically meaningful candidates. Comparable thresholds (7–10) are widely used in high-stringency proteomics and interactome studies.

The combined list of predicted targets was then screened for proteins potentially involved in glycosylation or colorectal cancer biology. Pathway identification and subsequent Gene Ontology and KEGG enrichment analyses were performed using the KEGG Pathway Database (https://www.genome.jp/kegg/pathway.html, accessed on 8 August 2025). Proteins lacking KEGG pathway assignments were further evaluated using WikiPathways (https://www.wikipathways.org/, accessed on 8 August 2025) to ensure comprehensive pathway coverage. A focused literature review was subsequently conducted to confirm associations with glycosylation processes and colorectal cancer. To ensure structural suitability for downstream docking, all shortlisted proteins were checked for the availability of high-quality 3D structures in the Protein Data Bank (https://www.rcsb.org/, accessed on 8 August 2025).

This integrative ligand-based workflow, combining ChEBI-derived structures, dual-platform target prediction, stringent score-based filtering, pathway enrichment, the literature validation, and structural verification, yielded a refined set of 15 high-confidence protein targets. These proteins represent colorectal cancer-relevant candidates supported by computational prediction and published evidence. Among them, protein kinase C-alpha (PrKCα) and mitogen-activated protein kinase 1 (MAPK1) emerged as the most plausible Brefeldin A interactors.

### 4.2. Computational Modelling Studies

#### 4.2.1. Preparation of MAPK1, PrKCα and Brefeldin A and Its Analogues

The 3D coordinates of cyclic adenosine 3′,5′-monophosphate (cAMP)-dependent protein kinase alpha (PrKCα; PDB ID: 3OVV, UniProtKB ID: P17612) were retrieved from the Protein Data Bank and processed using the Protein Preparation Wizard in Maestro v13.0. Energy minimisation was performed with the OPLS_2005 force field at physiological pH (7.0 ± 0.2). The stereochemical quality of the final energy-minimised structure was assessed with ProCheck, confirming that over 95% of residues occupied the most favoured regions of the Ramachandran plot. Similarly, the three-dimensional structure of Mitogen-Activated Protein Kinase 1 (MAPK1; UniProtKB ID: P28482) was obtained from the Protein Data Bank (PDB ID: 3SA0) and prepared using the Protein Preparation Wizard in Maestro v13.0. Energy minimisation was performed with the OPLS force field at physiological pH conditions (7.0 ± 0.2). The stereochemical integrity of the minimised structure was validated with ProCheck, which confirmed that more than 95% of residues were located within the most favoured regions of the Ramachandran plot.

A total of 51 Brefeldin A analogues (PubChem CID: 5287620) were retrieved from the PubChem database in 3D-SDF format and prepared using the LigPrep module in Maestro v13.0 with the OPLS_2005 force field for geometry optimisation and energy minimisation. This preparation step was essential to ensure that the ligands entered the docking and virtual screening pipeline in chemically stable, low-energy conformations with accurate stereochemistry, tautomeric states, and ionisation profiles. The Epik algorithm was applied to predict pKa values and generate relevant ionisation states at physiological pH (7.0 ± 0.2), followed by desalting and tautomer generation under the same pH conditions. For molecules with multiple chiral centres, stereoisomers were either retained or expanded as appropriate to yield chemically feasible low-energy structures. The processed ligands were stored in Maestro (.mae) format, yielding a final set of 43 ligands ready for high-throughput virtual screening (HTVS).

#### 4.2.2. Blind Docking of Brefeldin A into MAPK1 and PrKCα Using the AutoDock Vina Tool

Co-crystallised molecules considered irrelevant to protein kinase function, such as ions, crystallographic water, and non-essential ligands, were removed before docking analysis. Missing residues in the protein models, particularly in PrKCα, were reconstructed using the MODELLER algorithm [65] within the UCSF Chimaera GUI [51]. Ligand structures were prepared for docking using the Dock Prep tool in UCSF Chimaera. Brefeldin A was docked into the respective binding pockets of MAPK1 and PrKCα using AutoDock Vina v1.2, integrated within UCSF Chimaera [66], via the Dock Ligand tool (implemented in AutoDock Vina v1.2), which employs a flexible-body docking algorithm to predict ligand binding poses. Docking was performed under default parameters, including grid dimensions, search algorithms, and scoring functions. Binding affinities and docking poses were subsequently calculated, and the resulting conformations were visualised in Maestro and UCSF Chimaera. The top-ranked binding poses, corresponding to the most negative binding energies, were selected for MAPK1 and PrKCα, respectively. These complexes, (MAPK1:Brefeldin A and PrKCα:Brefeldin A) were prepared for molecular dynamics simulations to provide structural and dynamic insights into the inhibitory potential of Brefeldin A against the selected kinase.

#### 4.2.3. MM/GBSA-Based HTVS of Brefeldin A and Its Analogue, Targeting MAPK1 and PrKCα

Grid files for the MAPK1 and PrKCα receptors were generated in Maestro to initiate the high-throughput virtual screening (HTVS) workflow. The screening proceeded in three stages: HTVS, standard precision (SP) docking, and finally the extra precision (XP) protocol. At this stage, the MM/GBSA rescoring option was not applied. While Lipinski ADME filtering was omitted, QikProp filtering was incorporated during the HTVS step. The initial HTVS docking was performed on the full library of 43 Brefeldin A analogues, after which the top 10% of ligands were advanced to the SP-docking protocol. This tiered and systematic approach yielded a refined set of potential hits, which were subsequently evaluated and ranked based on Glide docking scores and Prime MM/GBSA calculations.

#### 4.2.4. Calculating Free Binding Energy Using MD Simulation Trajectories

The AMBER18 suite’s Graphical Processor Unit (GPU) version was used to run the MD simulation to calculate the binding affinity [67,68]. The investigated compounds were parameterised with the ANTECHAMBER module, which employed the bcc charge to generate atomic partial charges [69] (Gasteiger–Gaff) [70]. Topology and parameter files were generated with the LEAP module. Counter ions were added at a constant pH to neutralise the system, while a 20 Å TIP3P water box [71] was used to neutralise the system. After partially minimising the systems for 2500 steps with a 500 kcal/mol Å^2^ restraint potential, full minimisation for 5000 steps with no energy restraints was eventually implemented. Heating from 0 to 300 K was on the systems for 50 ps in an NVT canonical ensemble using a Langevin thermostat [72] and a harmonic restraint of 5 kcal/mol Å^2^. Equilibration was performed on the systems at 300 K for 1000 ps, while the ambient pressure was maintained at 1 bar using the Berendsen barostat, after which a 1000 ns MD production run was completed.

The thermodynamic binding free energy calculation is a method for probing the thermodynamic binding affinities of the various complexes, accounting for both enthalpic and entropic contributions. The Molecular Mechanics/Poisson–Boltzmann Surface Area (MM/PBSA) method was used to estimate the free energy of binding of small therapeutic molecules to biological macromolecules. This study computed the binding interaction free energy from the last 1000 ns trajectory. Mathematically, binding free energy is represented by the following equation:

(1) $$\Delta G_{\mathrm{bind}}=G_{\mathrm{complex}}-G_{\mathrm{receptor}}-G_{\mathrm{ligand}}$$

(2) $$\Delta G_{\mathrm{bind}}=\Delta E_{\mathrm{gas}}+\Delta G_{\mathrm{sol}}$$

(3) $$E_{\mathrm{gas}}=E_{\mathrm{vdW}}+E_{\mathrm{ele}}$$

A stringency factor of eight was initially employed to select prospective proteins of interest for further research.

(4) $$G_{\mathrm{sol}}=G_{\mathrm{PB}}+G_{\mathrm{SA}}$$

(5) $$G_{\mathrm{SA}}=\gamma \cdot \mathrm{SASA}\;$$

From the above equation, *E*_gas_ correlates with the gas-phase energy, whereas *E*_int_ denotes the internal energy. Coulomb and Van der Waals energies are denoted with *E*_ele_ and *E*_vdw_, respectively. In addition, *G*_sol_ is the free energy of solvation, whereas the polar solvation contribution is represented as *G*_PB,_ calculated using the following equation.

(6) $$∆G_{PB}=-\frac{1}{2}\;\left(1-\frac{1}{\epsilon_{out}}\right)\sum_{i,j}\frac{q_{i}q_{j}}{f_{PB}\left(r_{ij}R_{i}R_{j}\right)}$$

On the other hand, *G*_SA_ is the non-polar contribution, estimated from the solvent-accessible surface area (SASA) determined using a water probe of radius 1.4 Å with a surface tension constant, γ of 0.0072 kcal/(mol·Å^2^). For residue decomposition, analysis was also performed to determine the different energies each binding-site residue added to ligand affinity and stabilisation.

#### 4.2.5. Molecular Dynamics (MD) Simulation Studies

Molecular dynamics (MD) simulations were performed using a GPU-enabled Desmond engine implemented in Maestro v13.0. The complex corresponding to the top-scoring pose for Brefeldin A in complex with MAPK1 and PrKCα, and the respective apo forms of MAPK1 and PrKCα, were saved as PDB files and submitted to the Linux (Ubuntu) desktop server for the Desmond MD simulations studies. Before the MD simulation, each of the six systems (MAPK1_Apo, MAPK1_BrefA, MAPK1_BrefA-analogue, PrKCα_Apo, PrKCα_BrefA, and PrKCα_BrefA-analogue) was built using the System Builder module implemented in the Desmond algorithm. This solvated the system using the TIP3P explicit solvent model using the OPLS_2005 force field. The complex or apo protein was placed in an orthorhombic box (the distance from the box face to the outermost protein/ligand atom was set at 20 Å, and the box angle was *α* = *β* = *γ* = 90°).

The volume box containing the Brefeldin A or apo protein was minimised, and counter ions (placed at least 20 Å from each ligand) were added to neutralise the system. The system was physiologically conditioned by adding 0.15 M NaCl to the solvent box. After the solvation and ionisation phase in the explicit solvent model, the system was submitted to the molecular dynamics production phase. The MD simulation phase is divided into eight distinct stages, each with its own set of simulation parameters. Stages 1–7 constitute the equilibration, which comprises short simulation steps, while stage 8 is the final long-range 50 ns simulation stage. The type and parameters of the solvated system were detected in stage 1. In stage 2, a 100 ps simulation was performed using Brownian Dynamics under NVT conditions at 10 K with restraints placed on the solute-heavy atoms. Stage 3 involved a 12 ps simulation under NVT conditions at 10 K with restraints on heavy atoms. Stages 4, 6, and 7 (the pocket solvation in stage 5 was skipped) used short simulation steps (12, 12 and 24 ps, respectively) under NPT conditions (at 10 K and restraints on heavy atoms for stages 4 and 6). Stages 4, 6, and 7 (the pocket solvation in stage 5 was skipped) used short simulation steps (12, 12 and 24 ps, respectively) under NPT conditions (at 10 K and restraints on heavy atoms for stages 4 and 6). No restraints were placed on heavy atoms at stage 7. The final 1000 ns MD production stage at a constant temperature of 300 K and pressure (1 atm) was carried out at stage 8.

#### 4.2.6. Post-Dynamic Analysis

The molecular dynamics simulation-derived trajectories were viewed relative to post-dynamic analysis using Schrodinger Maestro v13.0 and GraphPad Prism for comparative analysis of protein structures. Succinctly put, both the simulation quality and the alpha carbon atoms’ root-mean-square deviation (RMSD) alongside the ligand with the receptor’s RMSD were assessed. In addition, the residue’s root-mean-square fluctuations (RMSF), secondary structure element analysis, and protein–ligand interaction analysis were performed using the simulation interaction diagram (SID) algorithm (implemented in Maestro v13.0). The radius of gyration (RoG) and atomic distance calculations were determined using the simulation events analysis algorithm (also implemented in Maestro v13.0).

### 4.3. Statistical Analysis

For each simulation condition, we generated time series of Cα-RMSD, radius of gyration (RoG), ligand RMSD (after fitting to the protein/pocket), and ligand SASA; trajectories were first superposed on protein Cα atoms to remove rotation/translation, equilibration frames were discarded, and values were sampled at ~1 ns intervals to limit autocorrelation (e.g., for MAPK1 under Apo, Brefeldin A, and Brefeldin A_analogue this yielded n ~ 1000 per group; N ~ 3000). Data were imported into GraphPad Prism v9/10 as a column table (one column per condition). Because distributions were non-Gaussian, we used non-parametric tests: Mann–Whitney U (two-tailed, 95% CI; large-N approximate *p*) for two-group comparisons and Kruskal–Wallis for ≥3 groups, followed, when significant, by Dunn’s multiple comparisons with Holm–Šidák adjustment. We report medians, IQRs, and Hodges–Lehmann median differences (with 95% CIs where applicable); GraphPad Prism outputs included U or H statistics and *p*-values, and results were visualised as box- and-whisker plots overlaid with individual points (Tukey format).

Finally, principal component analysis (PCA) was applied to evaluate the structural dynamics of PrKCα and MAPK1 enzymes, both in their apo states and under Brefeldin A treatment. The analysis involved parameters such as Cα-RMSD and RoG. This study used RStudio (Posit Software, PBC Version 2023.09.1+494) and the ggfortify package v0.4.19 [73,74], where the apo form is depicted in green, the Brefeldin A-treated form in red, and the Brefeldin A analogue-treated form in magenta. The PCA enabled a detailed examination of structural alterations induced by Brefeldin A, offering valuable insights into the dynamic responses of the protein to the transient binding of the ligands within the 1000 ns simulation time frame.

## 5. Conclusions

This study provides comprehensive molecular insights into the inhibitory potential of Brefeldin A and its analogues against MAPK1 and PrKCα, two key regulators of CRC progression and glycosylation-associated signalling. Using a combination of computational docking, molecular dynamics simulations, and free energy calculations, Brefeldin A and its analogues were shown to form stable interactions in the active sites of both kinases, inducing structural perturbations suggestive of functional inhibition. While Brefeldin A itself exhibited variable stability, particularly with MAPK1, its analogues demonstrated stronger and more consistent binding, highlighting opportunities for structural optimisation to enhance therapeutic efficacy. Additionally, the conformational dynamics analyses (RMSD, RMSF, RoG, PCA) revealed protein-specific effects, with MAPK1 showing moderate stabilisation and PrKCα displaying pronounced structural changes upon ligand binding. These findings are consistent with the known role of Brefeldin A in disrupting protein trafficking and glycosylation, reinforcing its potential as a dual inhibitor that can modulate glycosylation-related pathways critical for tumour development and survival. The moderate-to-high binding free energies further support the feasibility of developing Brefeldin A derivatives as kinase inhibitors.

Importantly, this work highlights glycosylation inhibition as a promising therapeutic strategy for CRC, a field with limited targeted options. Although these in silico results offer valuable mechanistic insight, further validation in vitro and in patient-derived models is essential to confirm BREFELDIN A’s biological efficacy and translational potential.

Collectively, this study not only lays a strong foundation for the repurposing of Brefeldin A but also underscores the value of computational approaches in accelerating the discovery and optimisation of inhibitors targeting glycosylation-associated proteins, thereby supporting future efforts in glycosylation-targeted drug development for colorectal and potentially other cancers.

## Figures and Tables

**Table 1:** Potential target proteins for Brefeldin A.

Brefeldin A Targets	Known Actives 2D (Topological Similarity) and 3D (Shape/Conformational Similarity)
CYP19A1 (Cytochrome P450 Family 19 Subfamily A Member 1)	55/69
ATP12A (ATPase H+/K+ Transporting Non-Gastric Alpha2 Subunit)	03/10
RPS6KA5 (Ribosomal Protein S6 Kinase A5)	04/03
AR (Androgen Receptor)	163/56
* PRKCα (Protein Kinase C Alpha)	49/203
PDCD4 (Programmed Cell Death Protein 4)	0/8
PGR (Progesterone Receptor)	31/40
TAS2R31 (Taste Receptor Type 2 Member 31)	0/1
PSEN2 (Presenilin 2)	66/0
PPARG (Perioxisome Proliferator-Activated Receptor Gamma)	12/10
JAK1 (Janus Kinase 1)	112/0
CDC25A (Cyclin-Dependent Kinase 2)	8/15
PDE10A (Phosphodiesterase 10A)	201/0
MAPK14 (Mitogen-Activated Protein Kinase Kinase 14)	118/0
* MAPK1 (Mitogen-Activated Protein Kinase 1)	133/0

* Two target proteins of interest, PrKCα and MAPK1, were identified as potential protein targets by manual curation and are indicated in the table above.

**Table 2:** KEGG pathway analysis for Brefeldin A target proteins.

Term Description	Matching Potential Target Proteins in the Network
Pathways in cancer	PPARG, **MAPK1**, AR, **PrKCα**, RPS6KA5, JAK1
Proteoglycans in cancer	**MAPK1**, PDCD4, MAPK1, **PrKCα**
PD-L1 expression and PD-1 checkpoint pathway in cancer	MAPK14, **MAPK1**, JAK1
MAPK1 signalling pathway	MAPK14, **MAPK1**, **PrKCα**, RPS6KA5
PI3K-Akt signalling pathway	**MAPK1**, **PrKCα**, JAK1
AGE-RAGE signalling pathway in diabetic complications	MAPK14, PrKCα

**Table 3:** Binding free energy decomposition of the Brefeldin A analogue in MAPK1 and PrKCα: The table presents calculated binding free energies (Δ*G*_bind_) and their component contributions, including Coulombic (Δ*G*_ele_), hydrogen bonding (Δ*G*_Hbond_), solvation (Δ*G*_sol_), and van der Waals (Δ*G*_vdW_) terms, along with the associated ligand strain energy. All values are expressed in kcal/mol. Results for MAPK1 show multiple binding poses, while PrKCα shows the dominant binding conformation. Negative values indicate favourable energetic contributions to binding, whereas positive ligand strain energies reflect conformational penalties incurred upon binding.

Protein	Brefeldin A Analogue	Δ*G*_Bind_ (kcal/mol)	Δ*G*_ele_ (kcal/mol)	Δ*G*_Hbond_ (kcal/mol)	Δ*G*_sol_ (kcal/mol)	Δ*G*_vdW_ (kcal/mol)	Lig_Strain_Energy (kcal/mol)
MAPK1	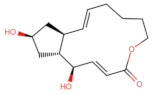	−42.33	−24.39	−1.57	33.80	−37.57	2.24
	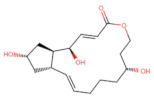	−38.35	−11.27	−1.37	17.95	−33.75	4.67
	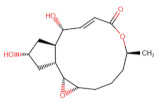	−33.70	−20.92	−2.19	25.81	−29.57	8.58
	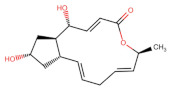	−37.35	−22.48	−2.24	26.00	−30.23	5.76
PrKCα	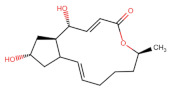	−37.82	−8.36	−1.54	21.87	−35.59	2.15

**Table 4:** MM/GBSA energy components (kcal/mol) for protein–ligand complexes computed from snapshots extracted over a 500 ns MD trajectory. Values are mean ± SD. Δ*E*_vdW_: van der Waals interaction energy; Δ*E*_ele_: electrostatic interaction energy; Δ*G*_gas_ = Δ*E*_vdW_ + Δ*E*_ele_ (gas-phase contribution); Δ*G*_sol_: implicit-solvent contribution (Generalised Born + surface area); Δ*G*_bind_ = Δ*G*_gas_ + Δ*G*_sol_ (no entropic term unless stated otherwise). More negative Δ*G*_bind_ indicates stronger predicted binding.

Systems	Energy Components (kcal/mol)				
	Δ*E*_vdW_	Δ*E*_ele_	Δ*G*_gas_	Δ*G*_sol_	Δ*G*_bind_
MAPK1_Bref_A_analog	−31.80 ± 3.08	−14.78 ± 7.20	−46.58 ± 7.18	17.76 ± 6.16	−28.83 ± 3.97
MAPK1_Bref_A	−37.31 ± 3.92	−19.24 ± 9.24	−56.56 ± 10.26	22.91 ± 7.13	−33.64 ± 5.54
PKCα_Bref_A_analog	−26.95 ± 2.85	−19.36 ± 8.00	−46.31 ± 8.33	26.45 ± 6.83	−19.86 ± 3.00
PKCα_Bref_A	−31.72 ± 5.19	−16.04 ± 12.59	−47.76 ± 15.57	23.71 ± 9.14	−24.05 ± 7.52

## Data Availability

The original contributions presented in this study are included in the article. Please direct further inquiries to the corresponding authors.
